# Depressive Symptoms and Self-Reported Emotion Regulation Strategy Use Among Empty-Nest Older Adults Following Recalled Happy and Sad Events

**DOI:** 10.3390/bs16060851

**Published:** 2026-05-26

**Authors:** Junni Wang, Jun Yang, Qianqian Zhang

**Affiliations:** School of Marxism, Xi’an University of Science and Technology, Xi’an 710600, China

**Keywords:** empty-nest older adults, depressive symptoms, recalled sad and happy events, self-reported emotion regulation

## Abstract

Although the psychological consequences of the empty-nest period are heterogeneous, depressive symptoms remain an important concern among empty-nest older adults in China. However, little is known about how depressive symptoms are associated with the self-reported use of emotion regulation (ER) strategies following positive and negative emotional events. This study compared the self-reported use of nine ER strategies following recalled happy and sad events among empty-nest older adults with high versus low depressive symptoms (*N* = 145) using generalized estimating equations. Older adults with higher depressive symptoms reported more frequent use of rumination and self-criticism following sad events, and more frequent use of cognitive reappraisal, expressive suppression, experiential avoidance, and self-criticism following happy events. They also reported less frequent problem-solving across both event types and less frequent acceptance and social sharing following happy events. In addition, they reported more frequent rumination following sad events and more frequent cognitive reappraisal and distraction following happy events, whereas the low-depressive-symptom group showed the reverse pattern. They also showed lower overall strategy-use ratings, a smaller strategy repertoire following sad events, and less differentiated repertoire patterns across happy and sad events. These findings provide descriptive evidence that depressive symptoms among empty-nest older adults are associated with distinct patterns of self-reported ER strategy use and repertoire size following recalled sad events.

## 1. Introduction

The psychological consequences of the empty-nest period vary across older adults, but depressive symptoms remain an important concern among some empty-nest older adults in China. Recent theoretical work suggests that the psychological outcomes of the empty-nest period are shaped by competing mechanisms of role loss and role strain relief, as well as broader cultural and social contextual factors (Hartanto et al., 2024). How older adults understand and cope with this transition may therefore be associated with their emotional well-being. For example, loneliness, role transitions, and loss of purpose following children’s departure can create stress and contribute to depression in some older adults (Mahakud et al., 2025; Park & Mendoza, 2022; L. J. Huang et al., 2019). Emotion regulation (ER) strategy use has been linked to how older adults cope with stress and to mental health differences (Jung, 2017; Guadalupe-Tixi & Santos-Morocho, 2024). Some strategies, such as suppression, avoidance, and rumination, have often been associated with poorer mental health, whereas others, such as reappraisal, problem-solving, and social sharing, have often been associated with better outcomes (L. Song et al., 2023; Ma & Song, 2023; H. Su et al., 2018; Dehkordi et al., 2025). However, these strategies may carry different psychological meanings across positive and negative emotional contexts. To date, few studies have systematically examined depression-related differences in empty-nest older adults’ use of multiple ER strategies across positive and negative emotional contexts. To address this gap, the present study compares self-reported ER strategy use between empty-nest older adults with high versus low depressive symptoms following recalled happy and sad events, thereby offering descriptive evidence on depression-related differences in strategy use in this population.

ER is commonly defined as the processes by which individuals influence which emotions they have, when they have them, and how they experience and express these emotions (Gross, 1998). Beyond the widely studied strategies of cognitive reappraisal and expressive suppression, prior strategy-based approaches have assessed a broader set of ER strategies, including acceptance, cognitive reappraisal, problem-solving, experiential avoidance, expressive suppression, self-criticism and rumination (Aldao & Nolen-Hoeksema, 2012; Dixon-Gordon et al., 2015). Importantly, broader strategy-based approaches provide a basis for selecting ER strategies that are relevant to specific populations and contexts (Aldao & Dixon-Gordon, 2014). For example, Gong et al. (2021) extended a prior seven-strategy set in their study of left-behind children by adding blaming others, aggression, and distraction, resulting in a ten-strategy framework. Building on this broader strategy-based perspective, the present study adapted the strategy set to the empty-nest context by retaining the seven commonly examined strategies and adding distraction and social sharing. Distraction has been widely considered relevant to managing depressive mood and has also been included in prior related ER research, whereas social sharing may be particularly relevant for empty-nest older adults because interpersonal sharing and social engagement have been associated with reduced depressive symptoms in this population (J. Su et al., 2022).

Despite growing attention to ER strategies, previous research has often focused on a limited set of commonly studied strategies, particularly cognitive reappraisal and expressive suppression, and has primarily examined young adults or general older adult populations. For example, Dai et al. (2014) examined older adults’ tendency to use reappraisal and expressive suppression, whereas other studies used forced-choice tasks, such as reappraisal versus distraction, to assess young adults’ strategy preferences across situations (Sheppes et al., 2011, 2014; Sang et al., 2018). Although these studies have advanced understanding of ER strategy use, binary or limited-strategy approaches may overlook how individuals report drawing on multiple strategies within emotionally meaningful contexts (Aldao, 2013). Given the changes in social roles and support resources that may accompany the empty-nest period, examining empty-nest older adults’ self-reported use of multiple strategies in response to specific emotional events may provide a clearer account of their perceived regulatory resources in the empty-nest context.

Prior research has largely focused on strategy selection in negative emotional contexts, such as anger, anxiety, sadness, and loneliness, with less attention to positive contexts such as happiness (Wang et al., 2026; Berkowitz et al., 2026; Dixon-Gordon et al., 2015; Gong et al., 2021). However, both positive and negative emotional experiences are relevant to later-life well-being, and difficulties in regulating both types of emotion have been linked to depressive symptoms (Xie et al., 2025). Less is known about whether empty-nest older adults with elevated depressive symptoms report different patterns of strategy use across positive and negative emotional contexts.

ER strategy use may vary across emotional contexts and depressive symptom levels, and the same strategy may carry different, or even contrasting, meanings in positive versus negative emotional contexts (Aldao, 2013; Bonanno & Burton, 2013). For example, cognitive reappraisal and problem-solving have often been associated with better adjustment in negative emotional contexts and are more frequently reported by individuals with lower depressive symptoms (Lennarz et al., 2019; Scheffel et al., 2019; Webb et al., 2012). However, their implications in positive emotional contexts appear less consistent. Some studies suggest that reappraisal and problem-solving in positive contexts may be associated with depressive symptoms (Berkowitz et al., 2026; Millgram et al., 2023), possibly because these strategies reflect cognitive processing that interferes with positive emotional experiences (Strauss et al., 2016; Veilleux et al., 2022), although findings on problem-solving remain mixed (Boemo et al., 2022; I. Mueller et al., 2024). Similarly, rumination is typically linked to depressive symptoms in negative contexts, whereas repetitive thinking about positive experiences may overlap with savoring or positive reminiscence and has been associated with better psychological functioning in some studies (Pacheco-Romero et al., 2024; Vanderlind et al., 2022). Acceptance and social sharing have also been widely discussed in relation to coping with negative emotions (Ford et al., 2018; Rimé, 2009), but their roles in positive emotional contexts remain less understood. These findings suggest the need to further examine the self-reported use of multiple strategies across positive and negative emotional contexts among empty-nest older adults with different levels of depressive symptoms.

Prior research has highlighted that depressive symptoms are associated with both strategy repertoire size and variation in strategy use across contextual demands. For example, individuals with elevated depressive symptoms tend to report smaller repertoires and greater difficulties in evaluating contextual demands and adjusting strategy use accordingly (Chen & Bonanno, 2021; X. Song et al., 2025). Wang et al. (2026) similarly found that empty-nest older adults with lower depressive symptoms reported larger strategy repertoires in sadness and anger contexts. However, because existing research has focused mainly on negative emotional contexts, less is known about positive–negative differences in strategy repertoire size and context-related variation in strategy use. In the present study, sadness and happiness were selected not only because both are relevant to depressive symptoms, but also because they represent emotionally contrasting events with opposing valence and potentially different regulatory goals. This positive–negative contrast may provide a more demanding and informative context for describing depression-related differences in strategy repertoire size and self-reported strategy use across emotionally distinct events among empty-nest older adults.

In summary, individuals’ depressive symptom levels may be associated with their patterns of strategy use across positive and negative emotional contexts, as well as with repertoire size and whether strategy use varies across contextual changes. However, existing evidence remains limited and somewhat inconsistent, especially regarding the use of multiple strategies across positive and negative emotional contexts among empty-nest older adults. Therefore, it is necessary to further examine whether empty-nest older adults with different levels of depressive symptoms differ in self-reported strategy use and repertoire size following happy and sad events.

The present study addresses this gap by comparing self-reported ER strategy use between empty-nest older adults with high versus low depressive symptoms following recalled sad and happy events. We proposed three hypotheses. First, compared with the low-depressive-symptom group, the high-depressive-symptom group would report more frequent use of strategies commonly associated with depressive symptoms, such as rumination and self-criticism following recalled sad events, and cognitive reappraisal, expressive suppression, experiential avoidance, and self-criticism following recalled happy events. Second, depression-related differences in strategy use would vary across recalled sad and happy events, such that the two groups would show different, and potentially contrasting, patterns of self-reported strategy use across these two event types. Third, the high-depressive-symptom group would report a smaller strategy repertoire and less differentiated repertoire patterns across recalled sad and happy events.

## 2. Methods

### 2.1. Participants

Participants were recruited using convenience sampling from several large older residential communities in western China. These communities were originally built in the 1970s and 1980s and included many older adults living apart from their adult children, making them suitable sites for recruiting empty-nest older adults who met the study criteria. A total of 228 empty-nest older adults were initially screened. Inclusion criteria were: age ≥ 60 years; living alone or only with a spouse and maintaining an empty-nest arrangement for >1 year; adequate communication ability; long-term Chinese residency; and no professional psychological treatment during the survey period.

Participants completed a sociodemographic questionnaire and several screening measures: the 10-item CES-D10, CSI-D, GAS-10, and Lawton IADL (Supplementary Materials). Based on established criteria (Q. B. Huang et al., 2015; G. Huang et al., 2020; Hall et al., 2000; A. E. Mueller et al., 2015; Mahoney & Barthel, 1965; Lawton & Brody, 1969), 16 participants were excluded (four withdrew; three changed living arrangement; two had neurological illness; one could not understand the questionnaire content; two reported being unable to recall any happy events; three had GAS-10 > 6; one had CSI-D ≤ 7). An additional 67 participants with moderate depressive symptoms (CES-D10 = 7–9) were not included in the subsequent study. The final sample comprised 81 in the low-depressive-symptom group (CES-D10 ≤ 6; age M ± SD = 72.41 ± 6.39) and 64 in the high-depressive-symptom group (CES-D10 ≥ 10; age M ± SD = 74.14 ± 6.52) among empty-nest older adults. The two groups did not differ significantly in age, sex, education, income, or chronic conditions (all *ps* > 0.05), but differed significantly in empty-nest type (χ^2^ = 6.984) and child support (χ^2^ = 42.767, both *ps* < 0.05), which were included as covariates in subsequent analyses. The study was approved by an institutional ethics committee. All participants provided written informed consent and received a small gift (≈30 RMB) upon completion.

### 2.2. ER Assessment

#### 2.2.1. Assessment Procedures

Based on prior research (Aldao & Nolen-Hoeksema, 2012; Dixon-Gordon et al., 2015), participants were asked to recall one specific sad event and one specific happy event that they had personally experienced, and then to report how often they had used each ER strategy in response to each recalled event. To reduce ambiguity in retrospective recall, participants were instructed to focus on the specific recalled event rather than on their general emotion regulation habits. Participants who were unable to recall either a happy or a sad event were excluded from the subsequent study.

The order of event recall was counterbalanced across participants, with some participants recalling the happy event first and others recalling the sad event first. The strategy questionnaire was administered in paper-and-pencil format.

#### 2.2.2. ER Strategies

Following established approaches (Aldao & Dixon-Gordon, 2014; Dixon-Gordon et al., 2015; Gong et al., 2021), the present study assessed the participants’ self-reported use of nine ER strategies following recalled happy and sad events. The nine strategies were acceptance (“allowing or accepting your feelings”), cognitive reappraisal (“thinking about the situation from a different perspective to change how you feel”), problem solving (“trying to change the situation or solve the problem”), expressive suppression (“hiding your feelings from others”), self-criticism (“criticize yourself for your feelings”), rumination (“repeatedly thinking about the situation that elicited the current emotion”), experiential avoidance (“push down your feelings or put them out of your mind”), distraction (“shifting your attention to something else”), and social sharing (“talking with close others about your feelings and seeking understanding”). For each recalled happy and sad event, participants rated the extent to which they used each strategy on a 4-point scale (0 = “not used at all”, 1 = “used a little”, 2 = “used some”, and 3 = “used very much”).

Similar to Dixon-Gordon et al. (2015), each ER strategy was assessed with a single item that provided a brief behavioral description of the strategy. The item wording served as the operational description of each strategy, and participants rated whether they had used the described response following each recalled event. Before completing the ratings, trained research assistants provided standardized instructions and checked whether participants understood the recall task and response scale. When participants had questions, research assistants clarified the task procedure or repeated the item wording but did not suggest answers or interpret the recalled events for participants. These single-item indicators were intended to capture brief, event-specific self-reported strategy use rather than comprehensive multi-item measures of each underlying construct. Therefore, internal consistency reliability, such as Cronbach’s alpha, was not calculated.

### 2.3. Data Analytic Plan

We used generalized estimating equation (GEE) models to examine how depressive symptom level, recalled event type, and ER strategy type relate to strategy use. GEE is appropriate for repeated-measures data with within-subject correlations and enables between-group comparisons while accounting for correlated observations (Hanley et al., 2003). We prioritized GEE because the primary goal was to estimate population-averaged (marginal) effects of group, event, and strategy type (and their interactions), rather than subject-specific effects that depend on random-effect parameters in mixed-effects models. Models specified a normal distribution with an identity link, an exchangeable working correlation structure, and robust (sandwich) standard errors, with participant ID as the clustering variable. Model fit was evaluated using QICC (corrected quasi-likelihood under independence model criterion), with smaller values indicating better relative fit.

Recalled event type and ER strategy type were within-subject factors, depressive symptom group was a between-subject factor, and strategy-use ratings served as the dependent variable.

Step 1 tested the main effects of event type (happy = 0, sad = 1), ER strategy type, and depressive symptom group (low = 0, high = 1) on overall strategy use. ER strategy type was entered as a categorical variable with nine levels: acceptance, cognitive reappraisal, problem solving, experiential avoidance, expressive suppression, self-criticism, rumination, distraction, and social sharing. For coding purposes, these nine strategy categories were coded from 0 to 8. These numerical values were used only as category labels and did not represent the number of strategies used. Strategy repertoire size was calculated separately as the number of strategies endorsed and therefore had a possible range of 0 to 9.

Step 2 added two-way and three-way interaction terms to examine whether strategy use varied as a function of group, event type, and ER strategy type. Significant interactions were followed up with pairwise comparisons using the Holm (sequential Bonferroni) adjustment.

Step 3 fitted nine strategy-specific GEE models (one per strategy) with each strategy’s ratings as the dependent variable and group, event, and their interaction (Group × Event) as predictors, to further characterize strategy-level interaction patterns. Planned contrasts within these models were also evaluated using the Holm (sequential Bonferroni) adjustment.

Step 4 evaluated group differences in repertoire size and variation in strategy use across event demands, using planned contrasts. Given the small number of a priori repertoire contrasts (four per threshold definition), *p* values for these contrasts were Bonferroni-adjusted for transparency.

Across all models, following prior research, empty-nest living type and child support were included as covariates (Zimmermann & Iwanski, 2014). Effects were evaluated using Wald χ^2^ statistics, and pseudo-R^2^ was reported as an effect-size index.

## 3. Results

### 3.1. Descriptive Statistics

**Figure 1:**
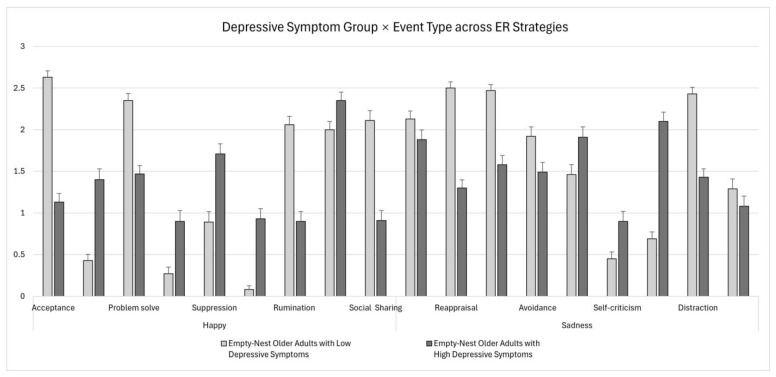
Estimated means from generalized estimating equations controlling for empty-nest living type and child support, with standard error bars.

Please see Figure 1 for the means and standard errors of ER strategies across participant groups and event types.

### 3.2. Primary Analyses

As shown in Table 1, the Step 1 GEE model revealed significant main effects of depressive symptom group, recalled event type, and ER strategy type. After the two-way and three-way interaction terms were added in Step 2, these main effects were further qualified by significant depressive symptom group × ER strategy type, recalled event type × ER strategy type, and depressive symptom group × recalled event type × ER strategy type interactions.

#### 3.2.1. Main Effects of Group, Event Type, and ER Strategy Type

Given the significant interaction effects, the main effects were interpreted as marginal patterns. Overall, the low-depressive-symptom group reported more frequent strategy use than the high-depressive-symptom group (B = 0.155, *p* < 0.001). Strategy use was also higher following recalled sad events than following recalled happy events (B = −0.252, *p* < 0.001). Compared with social sharing, acceptance, problem-solving, and distraction were reported more frequently, whereas experiential avoidance and self-criticism were reported less frequently (*ps* < 0.05).

In summary, older adults with higher depressive symptoms reported lower overall strategy use across recalled events and ER strategies.

#### 3.2.2. Interactions of Group, Event Type, and ER Strategy Type

The GEE model revealed significant two-way interactions between the depressive symptom group and ER strategy type, and between recalled event type and ER strategy type, as well as a significant three-way interaction among depressive symptom group, recalled event type, and ER strategy type (depressive symptom group × ER strategy type: Wald χ^2^(df = 8) = 172.37, *p* < 0.001; recalled event type × ER strategy type: Wald χ^2^(df = 8) = 197.24, *p* < 0.001; depressive symptom group × recalled event type × ER strategy type: Wald χ^2^(df = 8) = 190.81, *p* < 0.001). The depressive symptom group × recalled event type interaction was not significant (Wald χ^2^(df = 1) = 0.80, *p* = 0.37). Given these interaction effects, we conducted simple effects analyses to compare depressive symptom groups within each combination of ER strategy type and recalled event type.

Simple effects. All *p* values reported below are Holm-adjusted (sequential Bonferroni). Following recalled happy events, significant group differences were observed for eight of the nine ER strategies. Relative to the high-depressive-symptom group, the low-depressive-symptom group reported more frequent use of acceptance (Diff = 1.50, *p* < 0.001), problem-solving (Diff = 0.87, *p* < 0.001), rumination (Diff = 1.17, *p* < 0.001), and social sharing (Diff = 1.20, *p* < 0.001). Conversely, the high-depressive-symptom group reported more frequent use of cognitive reappraisal (Diff = −0.96, *p* < 0.001), expressive suppression (Diff = −0.82, *p* < 0.001), experiential avoidance (Diff = −0.62, *p* < 0.001), and self-criticism (Diff = −0.85, *p* < 0.001). The groups did not differ in distraction (*p* = 0.230).

Following recalled sad events, the high-depressive-symptom group reported more frequent use of rumination (Diff = −1.41, *p* < 0.001) and self-criticism (Diff = −0.45, *p* = 0.033), whereas the low-depressive-symptom group reported more frequent use of cognitive reappraisal (Diff = 1.19, *p* < 0.001), problem-solving (Diff = 0.89, *p* < 0.001), and distraction (Diff = 1.01, *p* < 0.001). No significant group differences were detected for acceptance, experiential avoidance, expressive suppression, or social sharing (*ps* ≥ 0.127).

### 3.3. Event-Related Group Differences Across ER Strategies

Planned contrasts indicated that group differences varied across recalled events and ER strategies (Table 2). All *p* values for planned contrasts were Holm-adjusted using the sequential Bonferroni procedure. Problem-solving showed a consistent group difference across events, with more frequent reported use in the low-depressive-symptom group following both recalled happy events (ΔHappy = 0.80, 95% CI [0.44, 1.16], *p* < 0.001) and recalled sad events (ΔSad = 0.82, 95% CI [0.43, 1.20], *p* < 0.001). No significant sad–happy change was observed within either group (ΔLow = 0.12, *p* = 0.428; ΔHigh = 0.11, *p* = 0.428).

Acceptance and social sharing showed event-specific group differences, characterized by larger Low–High differences following happy events that attenuated and became non-significant following sad events. For acceptance, the Low–High difference was significant following happy events (ΔHappy = 1.54, 95% CI [1.13, 1.95], *p* < 0.001), but not following sad events (ΔSad = 0.29, *p* = 0.076), accompanied by opposite-direction event effects within the two groups (ΔLow = −0.51, *p* < 0.001; ΔHigh = 0.75, *p* < 0.001). Social sharing showed a similar pattern (ΔHappy = 1.21, 95% CI [0.75, 1.67], *p* < 0.001; ΔSad = 0.21, *p* = 0.457; ΔLow = −0.83, *p* < 0.001).

Several strategies exhibited crossover interactions. For cognitive reappraisal, the high-depressive-symptom group reported more frequent use following happy events (ΔHappy = −0.86, 95% CI [−1.19, −0.53], *p* < 0.001), whereas the low-depressive-symptom group reported more frequent use following sad events (ΔSad = 1.30, 95% CI [0.96, 1.64], *p* < 0.001). This pattern corresponded to a marked increase from happy to sad events in the low-depressive-symptom group (ΔLow = 2.06, *p* < 0.001), but not in the high-depressive-symptom group (ΔHigh = −0.09, *p* = 0.583). Rumination showed a strong reversal (ΔHappy = 1.23, 95% CI [0.85, 1.62], *p* < 0.001; ΔSad = −1.34, 95% CI [−1.72, −0.96], *p* < 0.001), with opposite event effects between groups (ΔLow = −1.37, *p* < 0.001; ΔHigh = 1.20, *p* < 0.001). Distraction also showed a crossover pattern, shifting from a small difference favoring the high-depressive-symptom group following happy events (ΔHappy = −0.32, *p* = 0.045) to a larger difference favoring the low-depressive-symptom group following sad events (ΔSad = 1.03, 95% CI [0.70, 1.36], *p* < 0.001). This pattern reflected an increase in the low-depressive-symptom group (ΔLow = 0.43, *p* = 0.001) and a decrease in the high-depressive-symptom group (ΔHigh = −0.92, *p* < 0.001).

Overall, three group-by-event patterns emerged across ER strategies: problem-solving showed consistent group differences across events; acceptance and social sharing showed group differences only following happy events; and cognitive reappraisal, rumination, and distraction exhibited crossover interactions.

### 3.4. Strategy Repertoire Size and Event-Related Differences

All *p* values reported in this section are Bonferroni-adjusted. Repertoire size was defined as the number of strategies endorsed within each recalled event, with a possible range of 0 to 9. Because there is no universally established cutoff for determining whether a strategy should be counted as part of an individual’s ER repertoire, two operational criteria were used. Under the broad criterion, strategies rated greater than 0 were counted, indicating any reported use beyond “not used at all”. Under the strict criterion, only strategies rated 2 or higher were counted, indicating at least “used some” and excluding strategies rated only as “used a little”. Using both criteria allowed us to examine whether depression-related and event-related differences in repertoire size were consistent across a more inclusive and more conservative operationalization.

Using the strict endorsement criterion (rating ≥ 2), the low-depressive-symptom group reported a larger strategy repertoire than the high-depressive-symptom group following recalled sad events (Low–High = 0.67, 95% CI [0.04, 1.31], *p* = 0.032), whereas no significant group difference was observed following recalled happy events (Low–High = 0.56, *p* = 0.191). Both groups endorsed more strategies following recalled sad than happy events (Low: Sad–Happy = 1.01, 95% CI [0.60, 1.41], *p* < 0.001; High: Sad–Happy = 0.89, 95% CI [0.29, 1.49], *p* = 0.001). Using the broad endorsement criterion (rating > 0), no significant group differences emerged following either recalled happy or sad events (Happy: Δ = −0.58, *p* = 0.134; Sad: Δ = −0.08, *p* = 1.000). However, both groups again reported larger repertoires following recalled sad than happy events, with a greater sad–happy difference in the low-depressive-symptom group (Sad–Happy = 1.37, 95% CI [0.96, 1.78], *p* < 0.001) than in the high-depressive-symptom group (Sad–Happy = 0.87, 95% CI [0.35, 1.40], *p* < 0.001).

In summary, under the strict criterion, after recalling sad events, the low-depressive-symptom group reported a larger strategy repertoire than the high-depressive-symptom group, whereas no group difference was observed after recalling happy events. Under the broad criterion, the low-depressive-symptom group showed a larger difference in repertoire size between recalled sad and happy events than the high-depressive-symptom group.

## 4. Discussion

To our knowledge, this is among the first studies to examine the self-reported use of multiple ER strategies among empty-nest older adults with high versus low depressive symptoms following recalled happy and sad events. Across the nine strategies—acceptance, cognitive reappraisal, problem-solving, experiential avoidance, expressive suppression, self-criticism, rumination, distraction, and social sharing—the findings showed depression-related differences in reported strategy use that varied by recalled event type. Specifically, older adults with higher depressive symptoms reported more frequent use of several strategies commonly linked to depressive symptoms, a smaller strategy repertoire following recalled sad events, and less differentiated repertoire patterns across recalled sad and happy events. Overall, these findings provide a useful descriptive basis for understanding self-reported ER strategy use among empty-nest older adults with different levels of depressive symptoms.

### 4.1. ER Strategy Use Following Recalled Sad and Happy Events

The present study found that empty-nest older adults with higher depressive symptoms reported more frequent use of several ER strategies that have commonly been linked to depressive symptoms, which was generally consistent with Hypothesis 1. Specifically, they reported more frequent rumination and self-criticism following recalled sad events, and more frequent cognitive reappraisal, expressive suppression, experiential avoidance, and self-criticism following recalled happy events. These findings should be interpreted as differences in self-reported strategy use rather than as evidence that these strategies directly intensified sadness or dampened happiness.

Following recalled sad events, greater reported use of rumination and self-criticism may indicate more repetitive and negative self-evaluative responses among older adults with higher depressive symptoms. This interpretation is consistent with prior research linking rumination and self-criticism to depressive symptoms and prolonged negative affect (Gilbert, 2007; Watkins, 2008; Raes, 2010). Following recalled happy events, greater reported use of expressive suppression, experiential avoidance, and self-criticism may reflect less engagement with or expression of positive emotional experiences. However, because the present study did not assess regulatory goals, emotional outcomes, or changes in affect, these interpretations remain tentative. The interpretation of cognitive reappraisal following recalled happy events is discussed in greater detail in Section 4.2. Overall, these findings suggest depression-related differences in self-reported ER strategy use across recalled sad and happy events.

### 4.2. Event-Related Patterns of ER Strategy Use

The findings were generally consistent with Hypothesis 2, showing that depression-related differences in the self-reported use of ER strategies varied across recalled happy and sad events. First, empty-nest older adults with higher depressive symptoms reported less frequent use of problem-solving following both recalled event types. Problem-solving involves planning and active efforts to change or address a stressor (Billings & Moos, 1981), and may require greater behavioral engagement than primarily cognitive strategies such as reappraisal. This finding is consistent with prior evidence that older adults with higher depressive symptoms report less problem-solving in negative emotional contexts (Wang et al., 2026), and a similar pattern was also observed following recalled happy events in the present study. One possible interpretation is that elevated depressive symptoms may be associated with lower perceived capacity or motivation to engage in action-oriented responses across emotional events. However, because the present study did not assess regulatory goals, perceived controllability, or emotional outcomes, this interpretation should remain tentative.

Second, following recalled happy events, the high-depressive-symptom group reported less frequent use of acceptance and social sharing than the low-depressive-symptom group, whereas no group differences were found following recalled sad events. Acceptance has often been discussed in relation to coping with negative emotions (Ford et al., 2018), but it may also be relevant to remaining open to positive emotional experiences. Similarly, social sharing, traditionally examined in negative emotional contexts (Rimé, 2009), may also reflect interpersonal processing or elaboration of positive experiences. Thus, the lower reported use of acceptance and social sharing following happy events may suggest that older adults with higher depressive symptoms were less likely to engage with positive experiences or share them with others. This interpretation is consistent with interpersonal emotion regulation perspectives (Pruessner et al., 2020). However, the present findings should not be taken as evidence that these strategies were necessarily more effective or adaptive in the present study.

Finally, the two groups showed different event-related patterns for rumination, cognitive reappraisal, and distraction. Older adults with higher depressive symptoms reported more frequent rumination following recalled sad events, and more frequent cognitive reappraisal and distraction following recalled happy events. The low-depressive-symptom group showed the reverse pattern. Rather than indicating less adaptive or context-inappropriate strategy use, this pattern should be interpreted more cautiously as a difference in self-reported strategy-use profiles across recalled event types.

The greater reported use of cognitive reappraisal following recalled happy events among older adults with higher depressive symptoms is broadly consistent with prior evidence linking reappraisal in positive contexts to depressive symptoms (Berkowitz et al., 2026; Millgram et al., 2023). One possible interpretation is that reappraisal following positive events may involve cognitive processing that qualifies, downregulates, or dampens positive emotional experience (Strauss et al., 2016; Veilleux et al., 2022). Another possibility is that reappraisal reflects broader cognitive elaboration, meaning-making, gratitude, or efforts to place the positive event within a broader life context. Alternatively, this pattern may reflect ineffective use of reappraisal in positive emotional contexts. These interpretations remain tentative because the present study did not assess reappraisal content or emotional outcomes. Therefore, it cannot determine whether this pattern reflects positive-emotion dampening, ineffective reappraisal, or other forms of cognitive processing.

A similar caution applies to the interpretation of rumination and distraction. Repetitive thinking following recalled sad events may resemble rumination, whereas repetitive engagement with positive events may carry different meanings, such as savoring or positive reminiscence. Likewise, greater reported distraction following recalled happy events may suggest disengagement from positive emotional experience, but it may also reflect a general attentional shift rather than an attempt to avoid positive emotion. From the perspective of strategy–situation fit and emotion regulation flexibility (Aldao et al., 2015; Bonanno & Burton, 2013), these findings raise the possibility that depressive symptoms are associated with less differentiated or differently organized strategy-use patterns across emotionally distinct recalled events. Future studies should directly assess regulatory goals, perceived strategy fit, and emotional outcomes to clarify whether these patterns represent effective, ineffective, or event-specific forms of regulation.

### 4.3. ER Strategy Repertoire Across Recalled Events

The findings were generally consistent with Hypothesis 3, showing depression-related differences in strategy repertoire size following recalled sad events. Under the strict endorsement criterion, the low-depressive-symptom group reported a larger strategy repertoire than the high-depressive-symptom group following recalled sad events, whereas no group difference was found following recalled happy events. Under the broad endorsement criterion, both groups endorsed more strategies following recalled sad than happy events, but this event-related difference was larger in the low-depressive-symptom group. The low-depressive-symptom group also reported higher overall strategy-use ratings.

These findings are broadly consistent with prior work linking elevated depressive symptoms to smaller ER repertoires and lower flexibility-related capacities (Chen & Bonanno, 2021; X. Song et al., 2025). However, the present results should be interpreted as differences in self-reported repertoire size and event-related strategy-use patterns, rather than as direct evidence of reduced regulatory flexibility or lower context sensitivity. Notably, group differences in repertoire size were observed following recalled sad events but not following recalled happy events, suggesting that depression-related differences in repertoire breadth may be more evident when older adults recall negative emotional experiences. The absence of group differences following recalled happy events warrants further investigation.

These findings should also be interpreted within the Chinese sociocultural context. Because the present study was conducted among empty-nest older adults in China, cultural factors may shape how individuals cope with the empty-nest transition and how this transition relates to psychological well-being (Hartanto et al., 2024). In China, the empty-nest period is embedded in strong norms of family interdependence and filial responsibility. Children’s departure from the parental home may therefore be experienced not only as a residential transition, but also as a change in role identity, perceived family connectedness, and available interpersonal support. These cultural expectations may shape how older adults respond to emotional events and how they report using ER strategies. For example, expressive suppression and acceptance may be influenced by norms of emotional restraint and family harmony, whereas social sharing may depend on the availability of trusted family members or close community ties. Thus, the strategy-use patterns observed in the present study should not be directly generalized to Western populations, where norms surrounding later-life independence, parent–child relationships, emotional expression, and interpersonal sharing and support may differ.

## 5. Limitations

This study has several limitations. First, the cross-sectional design precludes causal inferences about the relationship between depressive symptoms and ER strategy use. Future longitudinal or experimental studies are needed to clarify directionality. Second, we acknowledge that the use of convenience sampling and an extreme-group design (excluding moderately symptomatic participants) may limit the generalizability of the findings and potentially inflate effect sizes. Therefore, our conclusions are intended to apply primarily to empty-nest older adults with relatively high- or low-depressive-symptom levels within similar community contexts. Future research should employ probability sampling and model depressive symptoms continuously or across multiple severity levels to validate and extend the present findings. Third, we did not manipulate or measure the intensity or recency of the recalled events to reduce cognitive burden in this older adult sample. Nevertheless, these factors may affect retrospective reports of strategy use. Future research should assess event intensity and recency or use experience sampling methods to address this limitation. Finally, each ER strategy was assessed with a single item. Although this approach provided brief indicators of self-reported strategy use, it may be limited in capturing the complex meanings of these strategies. Future studies should develop or use validated multi-item measures to more precisely assess the distinct constructs underlying each strategy.

## 6. Future Directions

Despite these limitations, the present study suggests several directions for future research. Prior work has suggested that individuals with depressive symptoms may report greater use of reappraisal and problem-solving in positive emotional contexts (I. Mueller et al., 2024). In contrast, empty-nest older adults with higher depressive symptoms in the present study reported more frequent cognitive reappraisal but less frequent problem-solving following recalled happy events. Future research should clarify the meanings of these strategies in positive emotional events, why social sharing is differently associated with depressive symptoms across positive and negative events, and why no group difference in strategy repertoire size emerged following recalled happy events.

## 7. Conclusions

This study found that depressive symptoms in empty-nest older adults were associated with distinct patterns in the self-reported use of ER strategy following recalled sad and happy events. Older adults with higher depressive symptoms reported more frequent use of rumination and self-criticism after sad events, and more frequent use of reappraisal, suppression, avoidance, and self-criticism after happy events, alongside less frequent problem-solving across both event types and less frequent acceptance and social sharing after happy events. They also reported a smaller strategy repertoire following recalled sad events and less differentiated repertoire patterns across recalled sad and happy events. These findings provide preliminary descriptive evidence on depression-related differences in ER strategy use and repertoire size among empty-nest older adults. They may also offer tentative reference points for identifying older adults’ perceived ER resources and support needs. However, given the cross-sectional and self-report design, the results should not be interpreted as causal evidence or as showing that changing strategy use would improve mental health outcomes.

## Figures and Tables

**Table 1:** GEE Wald χ^2^ tests and information criteria (QICC) for ER strategy use.

Predictor	Wald *χ*^2^	df	*p*	Pseudo *R*^2^ (%)
Step 1 (QICC: 2658.91)				
Empty-nest living type	0.14	1	0.71	0.02%
Child support	1.60	2	0.45	0.33%
Depressive symptom group	11.00	1	0.001	2.28%
Recalled event type	65.98	1	<0.001	13.65%
ER strategy type	404.62	8	<0.001	83.71%
Step 2 (QICC:2023.14)				
Empty-nest living type	0.14	1	0.71	0.01%
Child support	1.60	2	0.45	0.14%
Depressive symptom group	11.00	1	<0.001	0.98%
Recalled event type	62.09	1	<0.001	5.54%
ER strategy type	483.84	8	<0.001	43.20%
Depressive symptom group × Recalled event type	0.80	1	0.37	0.07%
Depressive symptom group × ER strategy type	172.37	8	<0.001	15.39%
Recalled event type × ER strategy type	197.24	8	<0.001	17.61%
Depressive symptom group × Recalled event type × ER strategy type	190.81	8	<0.001	17.04%

Note. Values are Wald *χ*^2^ statistics with corresponding df and p values. QICC = corrected quasi-likelihood under independence model criterion (smaller values indicate better relative fit). Pseudo *R*^2^ (%) denotes each predictor’s proportion of the total Wald *χ*^2^ within a given step and may not sum to 100% due to rounding. Models used an exchangeable working correlation structure with robust (sandwich) standard errors.

**Table 2:** Strategy-specific GEE Wald *χ*^2^ tests and QICC values by depressive symptom group and event.

Strategy	QICC	Empty-Nest Living Type Wald *χ*^2^ (*p*)	Child Support Wald *χ*^2^ (*p*)	Group Wald *χ*^2^ (*p*)	Event Wald *χ*^2^ (*p*)	Group × Event Wald *χ*^2^ (*p*)
Acceptance	201.43	0.04 (0.836)	3.36 (0.186)	48.58 (<0.001) **	1.90 (0.168)	50.45 (<0.001) **
Reappraisal	184.50	3.75 (0.053)	1.63 (0.443)	4.98 (0.026) *	96.25 (<0.001) **	115.46 (<0.001) **
Problem Solving	171.14	4.67 (0.031) *	15.56 (<0.001) **	43.47 (<0.001) **	2.08 (0.149)	0.01 (0.930)
Avoidance	245.90	1.05 (0.307)	13.51 (0.001) **	3.89 (0.048) *	128.17 (0 < 0.001) **	28.17 (<0.001) **
Suppression	324.09	3.36 (0.067)	0.45 (0.800)	15.45 (<0.001) **	13.76 (0 < 0.001) **	3.08 (0.079)
Self-criticism	181.33	2.41 (0.120)	0.55 (0.759)	27.91 (<0.001) **	4.74 (0.030) *	6.64 (0.010) *
Rumination	224.04	0.28 (0.600)	3.79 (0.150)	0.27 (0.606)	0.58 (0.446)	137.64 (<0.001) **
Distraction	192.38	2.30 (0.129)	2.93 (0.231)	14.20 (<0.001) **	6.59 (0.010) *	50.32 (<0.001) **
Social Support	304.85	0.84 (0.358)	1.49 (0.474)	26.36 (<0.001) **	9.43 (0.002) *	21.92 (<0.001) **

Note. Values are Wald *χ*^2^ statistics with *p* values in parentheses from strategy-specific GEE models. Each model included depressive symptom group, event (happy vs. sad), their interaction, and covariates (empty-nest living type and child support), with clustering by participant ID and an exchangeable working correlation structure using robust (sandwich) standard errors. QICC = corrected quasi-likelihood under the independence model criterion (smaller values indicate better relative fit). Degrees of freedom were 1 for all effects except child support (df = 2). ** *p* <0.001, * *p* <0.05.

## Data Availability

The data and analysis scripts are available at the Open Science Framework Repository https://osf.io/678ug (accessed on 8 April 2026).

## Supplementary Materials

The following supporting information can be downloaded at: https://www.mdpi.com/article/10.3390/bs16060851/s1, Questionnaire S1: The Center for Epidemiological Studies Depression Scale (CES-D10); Questionnaire S2: The Community Screening Interview for Dementia (CSI-D); Questionnaire S3: The 10-item Geriatric Anxiety Scale–Short Form (GAS-10); Questionnaire S4: Lawton Instrumental Activities of Daily Living Scale (Lawton-IADL).
