# Supplementary material for: Depressive Symptoms and Self-Reported Emotion Regulation Strategy Use Among Empty-Nest Older Adults Following Recalled Happy and Sad Events
Source: Behav Sci (Basel). 2026 May 26;16(6):851. doi: 10.3390/bs16060851 (PMC13296117; doi:10.3390/bs16060851)
Supplement: Supplementary file 1 [file behavsci-16-00851-s001.zip › behavsci-4281286-supplementary.pdf]

Supplementary materials for

Depressive Symptoms and Self-Reported Emotion Regulation Strategy Use Among Empty-Nest Older Adults Following Recalled Happy and Sad Events

Questionnaire used in the study

Questionnaire S1. the Centre for Epidemiological Studies Depression Scale (CES-D10)

Please rate according to your mood over the past week by checking the appropriate option. 0 = rarely or not at all, 1 = not much, 2 = sometimes or about half the time, 3 = most of the time.

1) I was bothered by things that don't usually don't bother me.

2) I had trouble keeping my mind on what I was doing.

3)I felt depressed.

4) I felt that everything I did was an effort.

*5) I felt hopeful about the future.

6) I felt fearful.

7) My sleep was restless.

*8) I felt happy.

9) I felt lonely.

10) I could not get "going".

Questionnaire S2. The Community Screening Interview for Dementia (CSI-D)

This questionnaire includes 7 items: Each correct answer is scored as 1 point. For item 7, recalling 1 word scores 1 point, 2 words score 2 points, and 3 words score 3 points. The maximum total score is 9. A total score of ≤7 is considered abnormal.

1) Naming the elbow

2) Stating the use of a hammer

3) Identifying the nearest market/shop

4) Identifying the day of the week

5) Identifying the current season

6) Pointing from the window to the door

7) Delayed recall of 3 words.

Questionnaire S3.The 10-item Geriatric Anxiety Scale–Short Form (GAS-10)

Please answer according to your actual feelings. 0 =not at all, 1 = sometimes, 2 =often, 3 =always. 0-6 points → indicates no anxiety, no need for concern.

1) I was irritable

2) I felt detached or isolated from others

3) I felt like I was in a daze

4) I had a hard time sitting still

5) I could not control my worry

6) I felt restless, keyed up, or on edge

7) I felt tired

8) My muscles were tense

9) I felt like I had no control over my life

10) I felt like something terrible was going to happen to me

Questionnaire S4. Lawton Instrumental Activities of Daily Living Scale (Lawton-IADL)

This scale includes 8 items. Each item is worth 1 point if performed independently. The maximum score is 5 for men and 8 for women. A higher score indicates greater independence in daily living activities and relatively better quality of life among older adults.

1) Ability to use telephone

2) Shopping

3) Food Preparation (for women)

4) Housekeeping (for women)

5) Laundry (for women)

6) Mode of Transporation

7) Responsibility for own Medications

8) Ability to Handle Finances
